# Draft Genome Sequence of the Nitrate- and Phosphate-Accumulating *Bacillus* sp. Strain MCC0008

**DOI:** 10.1128/genomeA.00189-12

**Published:** 2013-02-14

**Authors:** Shreya DebRoy, Amrita Bhattacharjee, Ashoke Ranjan Thakur, Shaon RayChaudhuri

**Affiliations:** a West Bengal University of Technology, Salt Lake, Kolkata, West Bengal, India; b Techno India University, Salt Lake, Kolkata, West Bengal, India

## Abstract

Here, we report the draft genome sequence of the nitrate- and phosphate-accumulating *Bacillus* sp. strain MCC0008, isolated from a consortium enriched from municipal sewage in nitrate broth (HiMedia M439). The total size of the genome is 5,609,456 bp, with a G+C content of 35.1%.

## GENOME ANNOUNCEMENT

Nitrate and phosphate are the two prime nutrients that are required of life because of their physiological processes. They are added as fertilizers to enhance the quality of soil, but only small fractions of them are utilized, resulting in their emergence as two of the most abundant pollutants in the world (1, 2). Nitrate and phosphate cause major problems to the health of humans and the environment, and thus their concentrations have to be maintained within certain limits (3). Irregular rainfall during different seasons and the streamflow pattern cause seepage of this contaminant from the soil to surface and groundwaters (4–8). Appropriate irrigation management is required for soils in order to avoid nitrate leaching. Sequestration of these essential nutrients is of utmost importance for agricultural reuse as plant growth nutrients. Some marine and nonmarine species of *Beggiatoa*, *Thioploca*, and *Thiomargarita* have shown nitrate accumulation in their intracellular vacuoles (9–11), while phosphate accumulation has been reported in many groups of bacteria. However, nitrate accumulators cannot be maintained as pure cultures for prolonged periods and, therefore, they cannot be used for routine sequestration of these nutrients. A *Bacillus* sp. strain, MCC0008, isolated from a consortium enriched from municipal sewage in nitrate broth (HiMedia M439), was found to accumulate nitrate and phosphate. Electron microscopy revealed vacuoles as are observed during nitrate accumulation in other genera. It also demonstrated phosphatase activity, as well as plant growth promotion, in the case of *Vigna radiata*.

The incomplete draft genome sequence of *Bacillus* sp. MCC0008 was obtained by sequencing on an Ion Torrent Personal Genome Machine (PGM) instrument on a 316 chip. The raw reads were used as input for *de novo* assembly using MIRA assembler v3.4.0. The total number of assembled reads was 1,740,538, the coverage being 43×. There were 331 contigs, of which 315 were large. A total of 307 Mb data were sequenced, with 202 Mb data with a quality value of >20. All contigs generated by MIRA assembly were submitted to the RAST (Rapid Annotation using Subsystem Technology) (12) server. The Mauve-based genome rearrangement analysis represents this as different from the three species of the *Bacillus* genus (*Bacillus anthracis, Baillus cereus*, and *Bacillus thuringiensis*). There are varying degrees of similarity among these three species at the metabolic level, as revealed by the RAST analysis.

The genes responsible for nitrate metabolism are the following: nitrite reductase [NAD(P)H] large subunit and small subunit, nitrate/nitrite transporter, and respiratory nitrate reductase alpha, beta, gamma, and delta chain genes are at GenBank accession no. ANAU01000016; the nitrate/nitrite sensor protein and the nitroreductase family protein genes are at GenBank accession no. ANAU01000036; and the nitrate/nitrite response regulator protein gene is at GenBank accession no. ANAU01000052. The genes responsible for YjbH-like GTP pyrophosphokinase domain, oligopeptide ABC transporter oligopeptide binding protein OppA, and alkaline phosphatase-like protein are at GenBank accession no. ANAU01000001; phosphate ABC transporter, periplasmic phosphate binding protein (PstS), and phosphate transport system permease proteins (PstC and PstA) are at GenBank accession no. ANAU01000020; phosphogycerate kinase and pyrophosphatase PpaX are at GenBank accession no. ANAU01000033; GTP pyrophosphokinase is at GenBank accession no. ANAU01000046; alkaline phosphatase-like protein is at GenBank accession no. ANAU01000062; and pX01-11 is at GenBank accession no. ANAU010000274. One hundred thirty-seven contigs code for plasmids, which are mostly reported in *Bacillus* sp.

### Nucleotide sequence accession numbers.

This Whole Genome Shotgun project has been deposited at DDBJ/EMBL/GenBank under the accession no. ANAU00000000. The version described in this article is the first version, ANAU01000000.
